# Getting more than what you pay for? Managing complications of bariatric tourism at an academic center near the US-Mexico border

**DOI:** 10.1007/s00464-025-11850-x

**Published:** 2025-06-05

**Authors:** Graham J. Spurzem, Patricia Ruiz-Cota, Amanda Rocha, Andres Fontaine-Nicola, Edgardo Reyes, Kiersten Gabaldon, Agustina Altolaguirre, Hannah M. Hollandsworth, Bryan J. Sandler, Santiago Horgan, Garth R. Jacobsen, Ryan C. Broderick

**Affiliations:** 1 Department of Surgery, Division of Minimally Invasive Surgery, University of California San Diego, 9300 Campus Point Dr., La Jolla, San Diego, CA 92037 USA; 2 University of California San Diego School of Medicine, San Diego, CA USA

**Keywords:** Medical tourism, Bariatric tourism, Bariatric surgery, Sleeve gastrectomy, Gastric bypass

## Abstract

**Background:**

Potential barriers exist for patients who desire bariatric surgery. Medical tourism, defined as international travel for the purpose of seeking medical care, has emerged as a popular alternative. Despite attempts at care standardization, substantial variation remains regarding institutional accreditation and the availability of appropriate postoperative bariatric care abroad. Management of postoperative complications therefore often falls to providers in the patient’s home country. We present our experience with the clinical and financial implications of bariatric tourism as an academic center located 30 miles from the US-Mexico border.

**Methods:**

A retrospective review of a prospectively maintained database identified patients who underwent cross-border bariatric surgery and then presented to our institution for management of postoperative complications from 2014 to 2024. Outcomes included type and number of procedural interventions required for complication management, length of stay (LOS), total intensive care unit (ICU) days, emergency department (ED) visits, readmissions, and mortality. Hospital charge and payment data for each patient were obtained, accounting for total LOS, interventions performed, readmissions, and ED visits.

**Results:**

A total of 91 patients were identified. The most common index procedure performed abroad was laparoscopic sleeve gastrectomy (*N* = 63, 69.2%). Common presenting complications included anastomotic/staple line leak (*N* = 30, 33.0%) and postoperative abdominal pain/nausea/vomiting (*N* = 24, 26.4%). In total, 194 procedural interventions were performed for complication management, including 112 upper endoscopies (57.7%) and 21 major surgical procedures (10.8%). 56.0% of patients required hospital admission on initial presentation and 19.8% required ICU admission. Anastomotic/staple line leak generated the highest mean hospital charges per patient ($424,975.89 ± $406,136.65), followed by enterocutaneous fistula ($277,076.50 ± $256,475.24). Overall mortality rate was 3.3% (*N* = 3).

**Conclusion:**

Bariatric tourism can present patients and local healthcare systems with significant clinical and financial challenges. Further studies are warranted to more comprehensively evaluate the implications of this practice.

Metabolic and bariatric surgery (MBS) is the most effective evidence-based treatment for obesity [1, 2]. Multiple studies with long-term follow-up have demonstrated that MBS provides superior sustained weight loss outcomes and reductions in obesity-related comorbidities compared to non-operative management [2–4]. The safety of MBS has also been established with low reported perioperative morbidity and mortality rates [5, 6]. MBS is gaining popularity as a result, with sleeve gastrectomy (SG) and Roux-en-Y gastric bypass (RYGB) being the most common procedures [7]. However, potential barriers exist for patients who desire bariatric surgery, including lack of insurance coverage, long waiting times, and preoperative approval protocols requiring demonstrated weight loss attempts and psychological evaluation [8, 9]. In response to these challenges, medical tourism, or international travel for the purpose of seeking medical care, has emerged as a popular alternative.

Population-based surveillance data indicate that millions of people each year travel outside of the United States (US) seeking various medical services, including cosmetic procedures, fertility treatment, and dental care [10]. It is estimated that at least 2% of worldwide bariatric procedures are performed for medical tourists, with Mexico being a top provider [11, 12]. Out-of-pocket costs for these procedures are often substantially cheaper than services provided in one’s home country [13]. A 2017 survey of bariatric surgeons worldwide found that the estimated mean cost of bariatric surgery in Mexico was $6400, compared to an average of $17,700 in the US [11]. Although bariatric tourism may allow some patients to circumvent barriers to obtaining care, the variability in accreditation procedures for bariatric centers internationally introduces substantial uncertainty with regard to patient safety, quality control, surgeon experience, and the availability of appropriate postoperative care [14]. There are currently no widely accepted international guidelines for the care of bariatric patients seeking surgery abroad and centers offering surgery to medical tourists operate under a variety of regulatory frameworks.

As the bariatric tourism industry expands, an increasing number of patients are returning to their home countries with a lack of structured postoperative care, incomplete documentation, and potentially life-threatening postoperative complications [15, 16]. Consequently, bariatric surgeons in the US are increasingly met with the challenge of managing these patients, who may experience complications requiring long-term medical and surgical care [17, 18]. As a tertiary care center located approximately 30 miles from the US-Mexico border, our institution frequently encounters patients presenting with complications following bariatric procedures performed in Mexico. In this study, we aim to present our experience with the clinical and financial implications of bariatric tourism and provide recommendations for safe postoperative follow-up and recovery of these patients.

## Methods

A retrospective review of a prospectively maintained database identified patients who underwent bariatric surgery in Mexico and then presented to our institution for management of postoperative complications from January 2014 to December 2024. Patient demographics included age, gender, Charlson Comorbidity Index (CCI), body mass index (BMI) at initial presentation, and type of insurance coverage. Data were also collected on the type of bariatric procedure performed abroad, type of presenting complication, and time from index operation to presentation at our institution.

Clinical outcomes included the type and number of procedural interventions required for complication management, hospital length of stay (LOS), total number of days spent in the intensive care unit (ICU), emergency department (ED) visits, hospital readmissions, total parenteral nutrition (TPN) requirement, and TPN duration.

To estimate the financial impact of these complications on our health system, all encounters including the initial presentation/admission and any subsequent hospital readmissions, ED visits, and outpatient procedures related to the bariatric complication were identified for each patient. Hospital charge and payment data were extracted for each of these encounters and totaled for each patient. These data account for the total cost of hospitalization, staffing, medical and procedural interventions, and material/supply costs. Continuous variables were reported as mean ± standard deviation (SD) or range. Categorical variables were reported as a frequency and percentage.

## Results

### Patient demographics & operative data

**Fig. 1:**
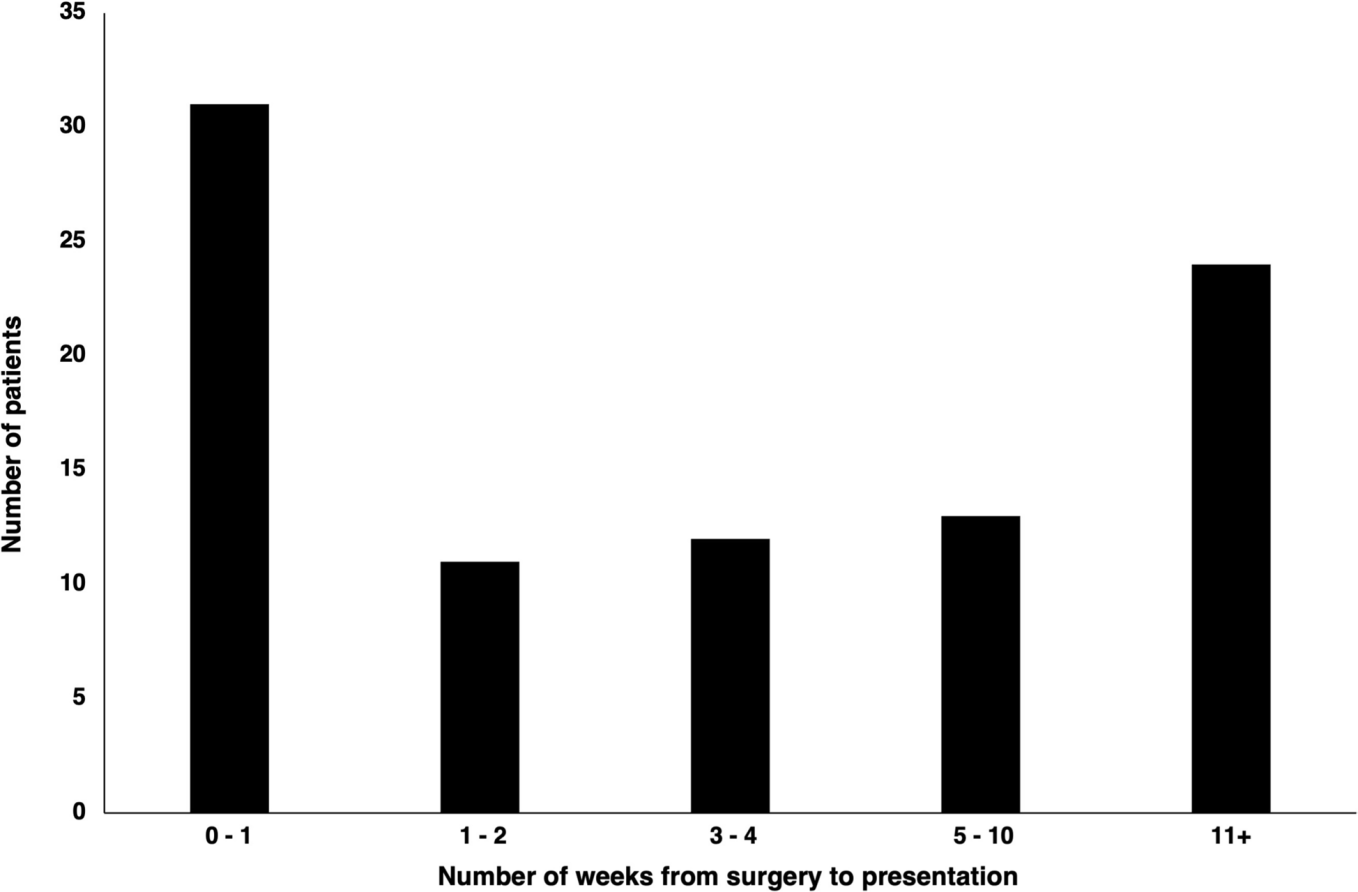
Time from index surgery in Mexico to presentation at our health system by number of weeks

A total of 91 patients who underwent bariatric surgery in Mexico and then presented to our institution were identified (Table 1). Mean patient age was 41.1 ± 11.9 years and most patients were female. Mean BMI at initial presentation was 39.3 ± 9.7 kg/m^2^. The most common procedure performed abroad was laparoscopic SG (*N* = 63, 69.2%) followed by laparoscopic RYGB (*N* = 7, 7.7%). The remaining patients underwent a variety of bariatric procedures, including revisions, conversions, intragastric balloon placement, and gastric banding. In terms of insurance coverage, most patients either had a private insurance plan (*N* = 39, 42.9%) or were enrolled in California’s Medicaid program known as Medi-Cal (*N* = 39, 42.9%). Many patients (*N* = 31, 34.1%) presented within 1 week after surgery, 12.1% (*N* = 11) between 1 and 2 weeks, 13.2% (*N* = 12) between 3 and 4 weeks, and 14.3% (*N* = 13) between 5 and 10 weeks (Fig. 1). The remaining patients presented more than 11 weeks after surgery, with some developing complications after several years.

**Table 1:** Patient demographics and operative data

Patient feature	All patients (*N* = 91)
Age (years), mean ± SD	41.1 ± 11.9
Female, N (%)	78 (85.7)
CCI, mean ± SD	0.60 ± 1.0
BMI at initial presentation (kg/m^2^), mean ± SD	39.3 ± 9.7
Type of insurance coverage, N (%)	
Medi-Cal	39 (42.9)
Medicare	2 (2.2)
Private	39 (42.9)
None	11 (12.1)
Index procedure performed abroad, N (%)	
Laparoscopic SG	63 (69.2)
Laparoscopic RYGB	7 (7.7)
Laparoscopic SG to RYGB conversion	3 (3.3)
Laparoscopic SG revision	2 (2.2)
Laparoscopic RYGB revision	1 (1.1)
Open SG	3 (3.3)
Intragastric balloon	4 (4.4)
Laparoscopic adjustable gastric banding	6 (6.6)
Laparoscopic BPD/DS	1 (1.1)
Laparoscopic vertical banded gastroplasty	1 (1.1)

*SD* standard deviation, *CCI* Charlson Comorbidity Index, *BMI* body mass index, *SG* sleeve gastrectomy, *RYGB* Roux-en-Y gastric bypass, *BPD/DS* biliopancreatic diversion with duodenal switch

### Postoperative complications

Patients presented with a range of complications with varying degrees of acuity (Table 2). The most common complication was anastomotic/staple line leak (*N* = 30, 33.0%), predominantly following laparoscopic SG. Several other complications requiring emergent intervention were also seen, including incarcerated port site hernias, missed enterotomy, perforated gastrojejunal (GJ) ulcer, small bowel obstruction, and an upper gastrointestinal bleed secondary to a gastric pouch ulcer. However, we also encountered several patients with some combination of postoperative abdominal pain, nausea, and vomiting (*N* = 24, 26.4%) for which our surgical service was consulted given the recent history of bariatric surgery. All of these patients had unremarkable cross-sectional imaging and were discharged from the emergency department after supportive care.

**Table 2:** Postoperative complications

Presenting complication, N (%)	All patients (*N* = 91)
Anastomotic/staple line leak	30 (33.0)
Laparoscopic SG	26
Laparoscopic SG revision	2
Laparoscopic RYGB revision	1
Laparoscopic BPD/DS	1
Postoperative abdominal pain/nausea/vomiting	24 (26.4)
GI stricture	5 (5.4)
Dysphagia	4 (4.4)
Port site hernia	3 (3.3)
Dehydration	2 (2.2)
Enterocutaneous fistula	2 (2.2)
Gastroesophageal reflux	2 (2.2)
Gastric outlet obstruction from balloon	2 (2.2)
Intra-abdominal hematoma	2 (2.2)
Venous thromboembolism	2 (2.2)
Portal vein thrombosis	1
Pulmonary embolism	1
Acute cholecystitis	1 (1.1)
Acute respiratory failure	1 (1.1)
Sepsis due to *Clostridium difficile* infection	1 (1.1)
Gastric band slippage	1 (1.1)
Gastric balloon pancreatitis	1 (1.1)
Hematuria	1 (1.1)
Incisional bleeding	1 (1.1)
Missed enterotomy	1 (1.1)
Perforated GJ ulcer	1 (1.1)
Small bowel obstruction	1 (1.1)
Starvation ketoacidosis	1 (1.1)
Superficial surgical site infection	1 (1.1)
Upper GI bleed secondary to gastric pouch ulcer	1 (1.1)

*SG* sleeve gastrectomy, *RYGB* Roux-en-Y gastric bypass, *BPD/DS* biliopancreatic diversion with duodenal switch, *GI* gastrointestinal, *GJ* gastrojejunal

### Clinical outcomes & interventions

In total, 194 interventions were performed for the management of postoperative complications, including esophagogastroduodenoscopy (EGD), major surgery, interventional radiology/pulmonology procedures, and TPN initiation (Table 3). The most common procedure was EGD with other interventions as indicated, such as stenting, clip placement, endo-vac placement/changes, and intragastric balloon removal (*N* = 122, 57.7%). A total of 21 major surgical interventions were performed, largely for emergent indications. In addition, 31 interventional procedures were performed by radiology and pulmonology, while a total of 31 patients required TPN initiation during their care for an average of 32.3 days (range: 1 – 132 days).

**Table 3:** Clinical outcomes and interventions performed for complication management

Intervention, N (%)	Total interventions (*N* = 194)
EGD with interventions (e.g. stent, clip, endo-vac, balloon removal)	112 (57.7)
Major surgical intervention	21 (10.8)
Laparoscopic gastric band removal	6
Exploratory laparotomy	6
Gastrectomy, RNY reconstruction	3
Abdominal washout, drain placement	1
Incisional hernia repair, resection of necrotic gastric remnant	1
Small bowel resection	1
Diagnostic laparoscopy	2
Laparoscopic incisional hernia repair	2
Laparoscopic abdominal washout, drain placement	1
Laparoscopic cholecystectomy	1
Laparoscopic resection of gastric perforation	1
Video-assisted thoracoscopic surgery, decortication	1
Interventional radiology & pulmonology	31 (16.0)
Peritoneal drain placement/exchange	28
Chest tube/thoracentesis	2
Bronchoscopy	1
TPN initiation	31 (16.0)

Outcomes, N (%) or mean (range)	Total patients (*N* = 91)
Hospital admission at initial presentation	51 (56.0)
Any ICU admission	18 (19.8)
Length of stay (days) for initial admission	16.0 (1 – 118)
ICU length of stay (days)	8.6 (2 – 28)
ED visits after initial presentation	1.2 (0 – 18)
Any readmission	24 (26.4)
TPN duration (days)	32.3 (1 – 132)
Final disposition outside of California	17 (18.7)
Mortality	3 (3.3)

*EGD* esophagogastroduodenoscopy, *RNY* Roux-en-Y, *TPN* total parenteral nutrition, *ICU* intensive care unit, *ED* emergency department

Among all patients, 51 (56.0%) required hospital admission at initial presentation with a mean hospital stay of 16.0 days. 18 patients (19.8%) required ICU admission at some point during their care with a mean ICU stay of 8.6 days. 24 patients (26.4%) were admitted to the hospital at least twice for management of their bariatric complication. Of note, 17 patients (18.7%) were discharged with a final disposition outside the state of California. Overall mortality rate was 3.3% (*N* = 3).

### Financial impact of complication management

For each patient, hospital charge and payment data were extracted for each encounter related to the management of their bariatric complication and totaled. Mean hospital charges, payments, and payment to charge ratios by presenting postoperative complication are detailed in Table 4. Mean hospital charges were highest for the management of anastomotic/staple line leak ($424,975.89 ± $406,136.65) and enterocutaneous fistula ($277,076.50 ± $256,475.24). The highest overall hospital charges were generated by the one patient who experienced a perforated GJ ulcer ($467,477.66). Patients who presented with some combination of postoperative abdominal pain, nausea, and vomiting had substantially lower mean hospital charges ($21,035.96 ± $10,076.44), as they were managed exclusively in the ED and discharged. While total hospital charges ranged widely depending on the complication, management of all 91 patients resulted in nearly $200,000 of hospital charges on average with an average payment to charge ratio of 0.27.

**Table 4:** Hospital charge and payment data by presenting complication

Presenting complication	Hospital charges, mean ± SD	Hospital payments, mean ± SD	Payment to charge ratio, mean ± SD
Anastomotic/staple line leak	$424,975.89 ± $406,136.65	$120,830.20 ± $148,491.89	0.30 ± 0.25
Postoperative abdominal pain/nausea/vomiting	$21,035.96 ± $10,076.44	$6,157.97 ± $7,210.95	0.29 ± 0.28
GI stricture	$246,057.75 ± $238,266.48	$32,094.05 ± $16,409.83	0.19 ± 0.15
Dysphagia	$71,451.29 ± $48,569.29	$18,582.92 ± $13,006.09	0.32 ± 0.21
Port site hernia	$179,964.39 ± $162,966.25	$15,369.40 ± $2481.54	0.14 ± 0.09
Dehydration	$11,322.93 ± $824.72	$3,634.00 ± $5,139.25	0.31 ± 0.43
Enterocutaneous fistula	$277,076.50 ± $256,475.24	$64,549.96 ± $52,755.67	0.25 ± 0.04
Gastroesophageal reflux	$28,771.62 ± $8919.43	$2,489.38 ± $2180.29	0.08 ± 0.05
Gastric outlet obstruction from balloon	$48,031.71 ± $11,138.30	$1,559.61 ± $632.15	0.03 ± 0.01
Intra-abdominal hematoma	$37,032.73 ± $25,133.01	$2,398.50 ± $3391.99	0.13 ± 0.18
Venous thromboembolism	$35,510.52 ± $32,237.52	$4,508.63 ± $2980.91	0.28 ± 0.34
Acute cholecystitis	$88,690.68	$50,206.85	0.57
Acute respiratory failure	$139,825.00	$50,394.00	0.36
Sepsis due to *Clostridium difficile* infection	$101,001.85	$9,760.02	0.10
Gastric band slippage	$9,167.84	$221.16	0.02
Gastric balloon pancreatitis	$41,898.44	$10,500	0.25
Hematuria	$18,084.54	$8,480.01	0.47
Incisional bleeding	$15,144.03	$9,808.02	0.65
Missed enterotomy	$90,048.58	$0	0.0
Perforated GJ ulcer	$467,477.66	$20,805.65	0.04
Small bowel obstruction	$137,652.52	$75,401.37	0.55
Starvation ketoacidosis	$28,570.40	$10,969.76	0.38
Superficial surgical site infection	$2,969.00	$192.78	0.06
Upper GI bleed secondary to gastric pouch ulcer	$73,936.75	$23,068.80	0.31
Total	$193,445.08 ± $297,415.92	$49,648.54 ± $98,776.71	0.27 ± 0.24

*GI* gastrointestinal, *GJ* gastrojejunal

## Discussion

With expansion of the bariatric tourism industry, surgeons in the US will increasingly be met with the challenge of managing patients who experience early and late complications following bariatric surgery abroad. Information regarding postoperative complications and associated hospital costs for these patients in the US are lacking. In this study, we report a series of 91 patients who underwent bariatric surgery in Mexico and then presented to our institution for complication management over a 10-year period. As an academic center located approximately 30 miles from the US-Mexico border, our health system is uniquely positioned to address the medical and surgical challenges of bariatric tourism. To our knowledge, this study represents the largest series of bariatric tourism complications and related financial implications in the literature.

We encountered a variety of postoperative complications with a wide range of clinical acuity. The most common complication was anastomotic/staple line leak, representing 33.0% of the tourism patients identified in this study, which is substantially higher than the estimated leak rate of less than 1% in the US based on national data [19]. While this rate is almost certainly an overestimation of the actual leak rate in Mexican centers due to the absence of a true denominator, it’s likely that US health systems can reasonably expect to encounter a high rate of serious complications such as anastomotic/staple line leak among tourism patients that present for care. Sheppard et al. studied the consequences of 62 tourism patients in Alberta, Canada and found the complication rate ranged from 42.2% to 56.1%, considerably higher than the local complication rate of 12.3% for bariatric surgery [17]. The authors also conservatively estimated the leak rate in the tourism group to be 43 times higher than patients who had surgery locally. While the actual denominator of patients engaging in bariatric tourism and therefore the true complication rate in this patient cohort is unknown, the high reported complication rates are likely exacerbated by the relatively short hospital stays these patients have at surgery centers abroad. A study of four high-volume bariatric centers in Tijuana, Mexico found that patients remained in the hospital for an average of 2.2 days postoperatively [20]. This likely results in patients returning to their home countries prior to the recognition of serious complications. US health systems are likely to encounter a large proportion of these potential postoperative complications given short hospital stays abroad and subsequent travel, which can introduce additional risk. However, we also encountered several less severe complications that did not require procedural intervention, including dehydration, minor incisional bleeding, and postoperative nausea/vomiting. The wide range of potential complications among bariatric tourists highlights the importance of postoperative management by experienced bariatric centers capable of performing the multitude of interventions that may be required for ongoing sequelae of these complications. This also underscores the importance for patients to establish postoperative follow-up with a local accredited bariatric surgery center upon returning home.

While postoperative complications can have potentially life altering consequences from a health standpoint, they can also result in a significant financial burden for patients. In our cohort, serious complications such as anastomotic/staple line leak, stricture, enterocutaneous fistula, and perforated GJ ulcer generated hundreds of thousands of dollars in hospital charges per patient. These costs can result in significant medical debt, even among insured patients [21]. According to a 2022 Kaiser Family Foundation report, an estimated 41% of adults in the US have some form of health care debt according to a broad definition including health care debt on credit cards or owed to family members [22]. The report also found that people with medical debt report cutting spending on food, clothing, and spending down savings to pay for medical bills. Tourism patients are particularly vulnerable to the impact of medical debt, as a common motivation for seeking care abroad is an inability to afford the same procedure at home. These complications can also have negative financial consequences for local health systems and public health insurance programs [23]. A survey of 25 Canadian surgeons reported an estimated 560,000 Canadian dollars spent on postoperative complication management for 59 bariatric tourism patients over a 1-year period [24]. Another Canadian study reported even higher costs for complication management after SG and RYGB [25]. Our analysis shows that the cost of complication management varies significantly depending on complication severity, with many patients requiring several costly procedural interventions over several months to years. The ratio of payments to hospital charges also varied considerably, which is dependent on several factors including insurance coverage [26]. Medicaid generally provides lower levels of reimbursement for services compared to fees paid by Medicare or private insurers [27]. Over 40% of patients in this study were insured by Medicaid, which potentially placed added financial strain on our health system in these cases due to lower reimbursement rates. Although the true impact of bariatric tourism complication costs on California’s Medicaid program is not clear, studies from the plastic surgery literature of complications from aesthetic procedures provide some insight. Khan et al. estimated that Medicaid pays about $730 million per year for complications from aesthetic procedures performed abroad [28]. Complications from bariatric tourism likely have similar consequences for our national health programs.

Given the potential risks associated with bariatric tourism, surgical societies have issued statements and recommendations with a primary concern for patient safety. The American Society for Metabolic and Bariatric Surgery (ASMBS) has issued several guidelines for global bariatric care [29]. If patients decide to seek care abroad, ASMBS recommends pursuing surgery at Joint Commission International (JCI) accredited centers, verifying surgeon credentials, obtaining complete medical records, and establishing a plan for postoperative follow-up at a local bariatric surgery program for longitudinal care. The American College of Surgeons (ACS) has issued similar recommendations for patients [30]. Surgical societies in Mexico have also attempted to establish consensus guidelines for the safe practice of bariatric tourism [12]. Despite this, a 2021 global survey of 383 bariatric surgeons worldwide by the International Federation for the Surgery of Obesity and Metabolic Disorders (IFSO) found that among surgeons who managed bariatric tourism patients, 24% of respondents stated they had no access to adequate documentation regarding a patient’s operation [15]. In addition, 12% of surgeons felt bariatric tourism was associated with higher mortality and only 49% felt that IFSO guidelines were followed by the foreign operating surgeon. Continued efforts are needed to encourage adherence to ASMBS and IFSO guidelines by international centers offering bariatric surgery, as a lack of information regarding a patient’s surgical care can be potentially detrimental to efforts by US surgeons tasked with managing complications. Some authors have advocated for the development and dissemination of discharge safety checklists to facilitate information transfer [31]. The development of national and international registries has also been proposed with the goal of tracking incoming and outgoing bariatric tourism patients in order to create a unified data source to track complications and inform patient decision-making [32].

Strategies aimed at educating patients regarding the inherent risks of bariatric tourism and which specific features to screen for at foreign centers may help to improve recognition of complications and reduce perioperative morbidity. Patients should be familiarized with the signs and symptoms of common postoperative complications to facilitate expeditious care. For example, patients should be aware that traveling after bariatric surgery can increase the risk of venous thromboembolism [33]. Depending on the country, there may also be a risk contracting infectious diseases endemic to different regions. Perhaps most importantly, patients should arrange for comprehensive bariatric care postoperatively in their home country that is covered by insurance and ensure the complete transfer of medical documentation. An analysis of websites from 34 international centers offering bariatric surgery found that only 67.7% of centers advertised some form of planned postoperative follow-up and just 14.7% provided a discharge summary to patients [14]. In light of this, patients should request complete medical records from foreign bariatric centers, including any preoperative workup, imaging, detailed operative notes, and any recommended postoperative care. Developing collaborations between domestic and nearby foreign accredited surgery centers may also help to facilitate the transfer of clinical expertise and information to optimize patient safety.

There are several limitations to this study, namely it’s retrospective, single-center design. Based on chart review, we were unable to accurately evaluate what proportion of patients had received appropriate postoperative instructions, documentation of their surgical procedure, or had a plan for postoperative follow-up in the US. We also were unable to identify the reasons patients decided to pursue surgery abroad, which would have provided important insight into this patient cohort. Our analysis of bariatric tourism complications was limited to patients who had surgery in Mexico, which may limit generalizability. In addition, the true cost of complications to patients and our health system was uncertain, as we were only able to obtain internal hospital charge/payment data as opposed to cost data, which is more complex to determine. An analysis of out-of-pocket expenses for patients experiencing these complications would be helpful to better define the potential impact of bariatric tourism on individuals and should be a focus of further studies. It should be emphasized that a major limitation of this study, and the study of bariatric tourism more broadly, is a lack of knowledge regarding the true number of patients who engage in this practice and their clinical outcomes. Thus, measurements and discussions of complication rates in this patient cohort should be interpreted with scrutiny. Further studies are warranted to more completely evaluate the clinical and financial impact of bariatric tourism complications on local and national health systems. Establishment of local and national patient registries may aid in these efforts.

## Conclusion

Bariatric tourism can present patients and local healthcare systems with significant clinical and financial challenges. Further studies are warranted to more comprehensively evaluate the implications of this practice.
